# Fungal Diversity in an Undisturbed Andean Páramo Soil in Quimsacocha (Ecuador)

**DOI:** 10.3390/jof10090623

**Published:** 2024-08-31

**Authors:** Ernesto Delgado-Fernández, Lidia Nicola, Sergio A. Covarrubias, Carolina Elena Girometta, Adrián Valdez-Tenezaca

**Affiliations:** 1 Laboratorios Ciencias de la Vida, Grupo de Investigación INBIAM, Departamento de Ingeniería Ambiental, Universidad Politécnica Salesiana, Calle Vieja 12-30 y Elia Liut, Cuenca 010102, Ecuador; mdelgado@ups.edu.ec; 2 Mycology Laboratory, Department of Earth and Environmental Sciences, University of Pavia, Via S. Epifanio 14, 27100 Pavia, Italy; carolinaelena.girometta@unipv.it; 3 Academic Unit of Chemical Sciences, Campus Siglo XXI, University of Zacatecas, Carretera Zacatecas-Guadalajara km 6, La Escondida, Zacatecas 98160, Mexico; sergio.hernandez@uaz.edu.mx; 4 Laboratorio de Patología Frutal, Departamento de Producción Agrícola, Facultad de Ciencias Agrarias, Universidad de Talca, Campus Talca, Av. Lircay s/n, Talca 360000, Chile

**Keywords:** Andes, metabarcoding, fungal diversity, environmental DNA, andean ecosystems microbiome vulnerable species

## Abstract

The Andean Páramo is an environment known for its high biodiversity; however, due to its remote location and difficult access, it is still relatively poorly studied. The aim of this work was to explore the fungal biodiversity of Ecuadorian Páramo soils in the undisturbed natural reserve of Quimsacocha through ITS metabarconding with an MiSeq platform. This analysis revealed the presence of 370 fungal Amplicon Sequence Variants (ASVs), mainly composed by *Ascomycota*, *Mortierellomycota* and *Basidiomycota*. The biodiversity had a great variability among the 19 samples, but the soil humidity proved to be a significant driver of diversity in the relatively dry environment of Páramo. Some of most abundant fungal genera have important relationships with plant roots. This work represents the first glimpse into the complex biodiversity of soil fungi in this understudied area, and further studies will be needed to better understand the fungal biodiversity in this region, together with the development of necessary measures of environmental protection.

## 1. Introduction

Fungi are essential organisms in the functioning of ecosystems, as they participate in the processes of decomposition, nutrient cycling, and symbiosis with other organisms. Andean Páramo soils are considered diverse and rich ecosystems for fungal species due to the high humidity and low temperature that characterizes these areas. High Andean forests are known for their high biodiversity, including fungal diversity, but due to their remote location and difficult access, they are relatively poorly studied. The results of recent studies reveal a high diversity of communities, with many species yet to be discovered and identified, with a high level of endemism [1,2,3]. Indeed, Barnes et al. [1] found a very high percentage of uncharacterized fungi, coupled with a high fungal beta diversity in their metabarcoding study of root-associated soil in the Bolivian Andes.

It is important to mention that most studies have focused on the identification of arbuscular mycorrhizal fungi and the description of new species [4,5,6]. One study, for example, explored the diversity and structure of arbuscular mycorrhizal communities in the high Andean forests of Ecuador and found that the composition of these communities were influenced by environmental factors such as nitrogen and phosphorus concentration [7]. Therefore, there is a need for broader and more systematic studies to understand fungal diversity in these soils and their relationship with other environmental factors.

Quimsacocha is a natural reserve located in the province of Azuay, Ecuador, known for its great importance in the conservation of biodiversity and the provision of ecosystem services. The area hosts a great diversity of flora and fauna species, with over 500 plant species, 53 mammal species, 149 bird species, and 22 amphibian and reptile species being registered there. The reserve is a refuge for an important variety of endemic and endangered species [8,9]. Additionally, the area is an important source of water for nearby populations and the city of Cuenca, Ecuador. The reserve also plays a crucial role in climate regulation, soil protection, and the prevention of natural disasters [10,11].

The aim of this study was exploring soil fungal diversity in the Andean Páramo of Quimsacocha. It provides valuable information for future research regarding the conservation and management of this type of ecosystem. Therefore, this information on fungal diversity in Andean Páramo soils is not only important for understanding the ecology and biology of these organisms in the context of biodiversity conservation but also for understanding how these ecosystems could be affected by climate change and other disturbance processes.

## 2. Materials and Methods

### 2.1. Sampling Site and Collection

**Figure 1:**
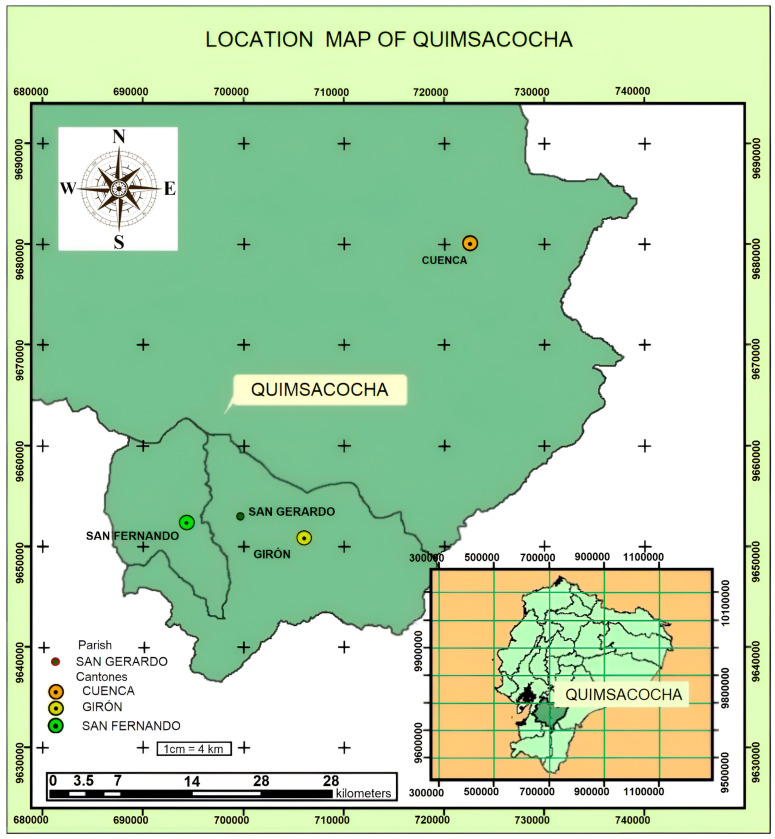
The upper part shows the location of the Quimsacocha sector within the area that corresponds to the cantons of Cuenca, Giron, San Fernando, and the province of Azuay, Ecuador. The lower region shows a map of Ecuador and the location of the province of Azuay.

The study site was located on the Quimsacocha reserve, Ecuador, at elevations that vary from 3040 to 3960 m.a.s.l., west of the Andes Mountains (Figure 1), 30 km southwest of the city of Cuenca, Azuay. The area consists of three sectors, Cristal, Cerro Casco and Rio Falso. These last two sectors are located within the “YanuncayIrquis” forest reserve and the Cristal sector, which is located within the “El Chorro” forest reserve, an area with an extension of 7960 ha which is located at the UTM SAD 56 coordinates 698,750 E, 9,663,400 N. The average annual rainfall of this region ranges between 1060 mm and 1600 mm per year [12]. The flora surrounding the study site is Páramo, typically dominated by tussock plants, acaulescent rosettes, and erect and prostrate herbs as a result of the Andean environmental conditions, such as the strong variability of rainfall, wind, and temperatures. The tussock Páramo at this site is dominated by bunch-grasses of the genera *Stipa* and *Calamagrostis* (both *Poaceae*). Other recorded abundant genera with various species include *Hypericaceae* (*Hypericum*), *Poaceae* (*Paspalum, Cortaderia*), *Caprifoliaceae* (*Valeriana*), *Asteraceae* (*Baccharis, Chuquiraga, Gynoxys, Diplostephium, Werneria, Loricaria*), *Apiaceae* (*Eryngium*), *Araliaceae* (*Hydrocotyle*), *Gentianaceae* (*Gentianella*, *Halenia*), *Geraniaceae* (*Geranium*), *Lycopodiaceae* (*Huperzia*), *Pteridaceae* (*Jamesonia*), *Fabaceae* (*Lupinus*), *Campanulaceae* (*Lysipomia*) and *Bromeliaceae* (*Puya*). Cushion forming *Plantaginaceae* (mostly *Plantago rigida*) and *Cyperaceae* (*Carex*) are typically found in moist depressions of higher elevated Páramo sites [13].

Quimsacocha was divided into three altitudinal levels (L1, L2, L3; Table 1), and in October 2018, 25, 25 and 27 soil samples were taken for each level, making a total of 77 soil samples. Each sampling point was selected by a completely random design in an area of 10 m^2^. The number of samples per altitude level fully covered the extension of the altitude level. Moreover, during the sampling, the presence of water sources was taken into account, such as the proximity of sampling sites to little streams and swampy sites with a visible presence of high humidity. Using a manual shovel, soil cores (10 cm long × 10 cm wide × 20 cm deep) were taken, and the shovel was disinfected with 80% ethanol after each sampling. Then, the soil samples were placed in a cooler with ice for conservation, transported to the laboratory, and stored at 4 °C. Subsequently, insects, rocks, and plant remains were removed and the samples were sieved at 2 mm and stored at −20 °C (for environmental DNA extraction) and metagenomic analysis.

### 2.2. Physicochemical Analysis of Soils

Soil physicochemical analyses were performed on soil samples from each sampling altitudinal level (L1, L2, L3) following the methods reported by Bloem et al. [14]. From each area (L1, L2, L3), three replicates were prepared by grouping 100 g aliquots derived from each soil sample; each grouped sample was then sieved (2 mm mesh) and homogenized according to the method reported by Uroz et al. [15]. Hydrogen potential was measured with an INESA pH meter (Shanghai REX Instrument Factory, Shanghai, China) in a 1:5 suspension of ultrapure water. Organic matter was evaluated following the Walkley–Black method [16]. The elements sodium, iron, zinc, manganese, copper, sulfur, calcium, magnesium, potassium, chlorine, and phosphorus were measured using inductively coupled plasma atomic emission spectrophotometry (ICP-AES), boron by ion chromatography, and nitrates by volumetric titration Kjeldahl. Each measurement was made in triplicate and the mean for each sampling site was reported. The samples were harvested from clay and sandy soils in Quimsacocha. The moisture content of each location where the soil samples were taken was measured using a rod hygrometer at a depth of 15 cm. Three measurements were taken for each sample.

### 2.3. Extraction of Environmental DNA in Soil Samples

Environmental DNA was extracted and purified from 250 mg of soil from each of the 77 samples using the DNeasyPowerSoil Pro Kit isolation kit (Qiagen, Hilden, Germany) according to the manufacturer’s instructions, as it has been shown to be a robust method for DNA extraction from soils [17]. The DNA samples were pooled in sets of 4 neighboring samples (coming from 4 sampling areas that had at least two contigouous sides among each other), generating a total of 19 environmental genomic DNA samples, which were used to analyze fungal populations. The quality and size of the DNA was verified by 2% agarose gel electrophoresis. The additional control of the quality of the extracted environmental DNA was carried out using a Thermo Scientific Nanodrop 2000 Photometer Spectrometer, measuring 260/280 and 260/230 ratios. DNA concentration was determined using Qubit Fluorometric Quantitation (Life Technologies, Carlsbad, CA, USA).

### 2.4. PCR Amplification and Next-Generation Sequencing (NGS, Illumina MiSeq)

Polymerase chain reaction (PCR) amplification of ITS hypervariable regions (6F-4R) was performed using the purified environmental DNA as a template to amplify an internal fragment of the ITS region. The primers ITS 3 (5′-GCA TCG ATG AAG AAC GCA GC-3′) and ITS 4 (5′-TCC TCC GCT TAT TGA TAT GC-3′) [18] were linked to a multiple identifier sequence (Illumina) following standard procedures recommended by the manufacturer [19]. Amplicons were generated for each sample in several duplicate PCRs using mixes (25 μL) containing 25 pmol of each primer, 1x KAPA HiFiHotstart Ready Mix (Kapa Biosystems, Wilmington, MA, USA), and 15 ng of template DNA. The PCR program consisted of an initial denaturation step at 95 °C for 3 min, 25 cycles of denaturation at 95 °C for 30 s, primer annealing at 55 °C for 30 s, and an extension at 72 °C for 30 s, followed by a final heating step at 72 °C for 5 min. Amplicons from the same treatment were pooled to reduce variability by PCR and purified using AMPure XP beads (Beckman Coulter, Brea, CA, USA) according to the manufacturer’s instructions. After PCR cleanup, Illumina sequencing adapters were joined by a second stage PCR using the Nextera XT Index Kit (Illumina Inc., Sand Diego, CA, USA). The mix contained Nextera Index Primers 1 and 2 (5 µL), 2 KAPA HiFiHotstartReadyMix (25 µL), DNA (5 µL), and PCR-grade water (10 µL) for a total volume of 50 µL. The PCR program at this stage consisted of an initial denaturation process at 95 °C for 3 min, followed by 8 cycles of denaturation at 95 °C for 30 s, a primer annealing at 55 °C for 30 s, an extension at 72 °C for 30 s, and a final step at 72 °C for 5 min. Amplicons were cleaned as described above. Amplicon libraries were quantified using Qubit (Invitrogen, Waltham, MA, USA). Fragment sizes were checked and template size distribution was verified using an Agilent Technologies 2100 bioanalyzer with a DNA 1000 chip. Samples were sequenced on a MiSeq platform (2 × 300 paired-end sequencing, considering 2 × 50,000 reads/sample) from Illumina at Macrogen (Seoul, Republic of Korea) according to the manufacturer’s instructions.

### 2.5. Taxonomic Assignment of Sequence Reads and Diversity Indices

Paired-end read sequences generated from the Illumina MiSeq were processed using the “Quantitative Insights Into Microbial Ecology 2” (QIIME 2, v2018.6) software package [20]. Briefly, reads from the ends of the demultiplexed pair were trimmed, filtered, and fused with the DADA2 complement [21], maintaining sequences with a minimum quality score of 25, a minimum length of 240 bp for reads reverse, and a maximum length of 260 bp for advanced reads. The merged reads were collapsed into representative sequences or Amplicon Sequence Variants (ASVs); then, the ASVs were chimera-filtered de novo using VSEARCH [22]. Sequences were then filtered for singletons and doublets (sequences are observed only once or twice). The taxonomy of the ASVs was assigned a 99% sequence identity based on the UNITE v7 database [23]. Non-fungal sequences were removed from further analysis, and the ASV table was thinned to a uniform depth (100,000 sequences per sample) to reduce biases related to sequencing depth.

The taxonomy and shared files produced in Qiime were imported into R [24], using the Phyloseq package version 1.44.0 [25], where the diversity indices of Shannon, Chao1 and Simpson and the number of ASVs were calculated. The ASVs and taxa data are presented as relative abundance percentages, calculated as the number of reads found for one ASV/taxon in a sample against the total number of reads detected in that sample. The average abundance of a taxon/ASV in the Quimsacocha area was calculated as the mean of the relative abundances in all the samples for that taxon. The alpha diversity was calculated using observed species and Shannon index estimates. β diversity was estimated based on the Bray–Curtis distance matrix used to calculate the principal coordinate plot (PCoA). The statistical test PERMANOVA was used, implemented in the vegan R package, ver. 2.5.6 [26], to assess any statistically significant differences among the fungal communities in the different sampling areas.

### 2.6. Access Numbers

High-throughput sequencing data sets were deposited in the NCBI Biosamples database under accession numbers SAMN28020743, SAMN28020744, and SAMN28020745 for Altitudinal Level 1, Altitudinal Level 2, and Altitudinal Level 3 for ITS DNA metabarcoding libraries, respectively.

## 3. Results

### 3.1. Soil Physico-Chemical Analyses

The soils in the three altitudinal levels were all slightly acidic (pH < 5.5). The soils in L1 had the highest organic matter content, P, Zn and Fe, while the soils in L3 had the highest content in NH_4_, S, Cu, and Mn when compared to the other locations (Table 2).

Moreover, the moisture of soil samples was measured, and the samples were divided into two groups (dry and wet) according to the level of moisture (Table 3). Dry sampling sites had an average water content lower than 55% and no proximity to visible water sources, while wet sampling sites had an average water content above 65% and were in proximity to water sources, such as little streams or swamps.

### 3.2. Soil Fungal Assemblage Composition

**Figure 2:**
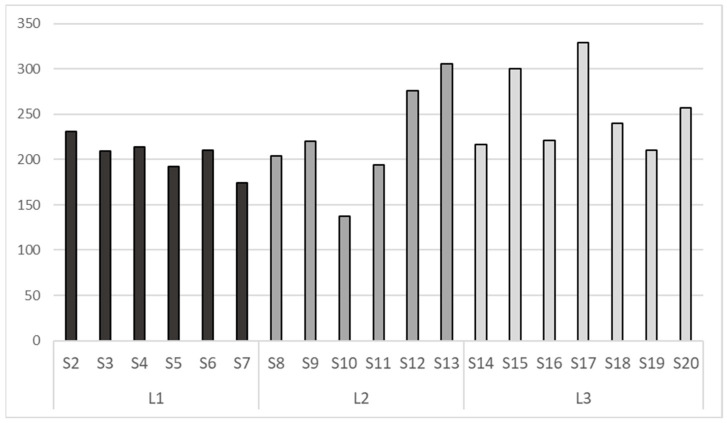
Number of ASVs present in each sample, with S2, S3, S4, S5, S6, S7 belonging to altitudinal level L1; S8, S9, S10, S11, S12, S13 belonging to L2, and S14, S15, S16, S17, S18, S19, S20 belonging to L3.

The sequencing on the Illumina Miseq Platform of the DNA of the 19 pooled soil samples produced a total of 1,018,335 reads, with an average of 53,597 reads per sample. After the cutting, trimming, and filtering process, the reads were reduced to 506,435, with an average of 26,654 reads per sample. It was observed that the “Level 3” altitude showed the maximum abundance with an average of 254 ASVs (Amplicon Sequence Variants), followed by “Level 2” with an average of 223 ASVs and “Level 1” with 205 ASVs (Figure 2).

**Figure 3:**
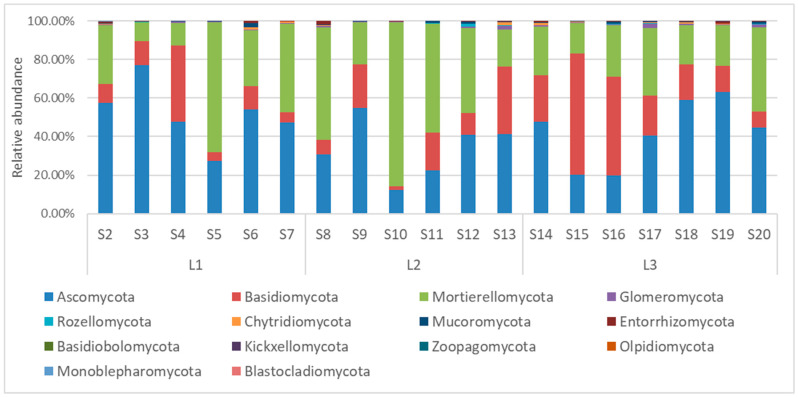
Soil fungal relative abundances at the phylum level of soil samples taken from the Quimsacocha reserve, Ecuador.

Metabarcoding data were taxonomically organized in this work following the high-level classification of the fungi reported by Tedersoo et al. [27]. In these samples, a total of the following 14 different fungal phyla were detected: *Ascomycota*, *Basidiomycota*, *Mortierellomycota*, *Glomeromycota*, *Rozellomycota*, *Chytridiomycota*, *Mucoromycota*, *Entorrhizomycota*, *Basidiobolomycota*, *Kickxellomycota*, *Zoopagomycota*, *Olpidiomycota*, *Monoblepharomycota*, and *Blastocladiomycota* (Figure 3). The composition of the fungal communities, even at the phylum level, was different among the samples. *Ascomycota* was the most abundant phylum, with an average abundance of 42.57%, ranging from 12.41% in S10 to 77.20% in S3. The second most abundant phylum was *Mortierellomycota*, with an average abundance of 35.16%, ranging from 10.08% in S3 to 85.42% in S10. *Basidiomycota* followed with a lower abundance (average abundance 20.07%, ranging from 1.61% in S10 to 62.71% in S15). Other phyla had an average abundance comprised between 0.1% and 1%, and they were were *Glomeromycota* (0.69%), *Mucoromycota* (0.54%), *Entorrhizomycota* (0.40%), *Chytridiomycota* (0.36%), and *Rozellomycota* (0.19%). All the remaining phyla had an abundance lower than 0.1%.

#### 3.2.1. Subkingdom Dikarya

##### Ascomycota

**Figure 4:**
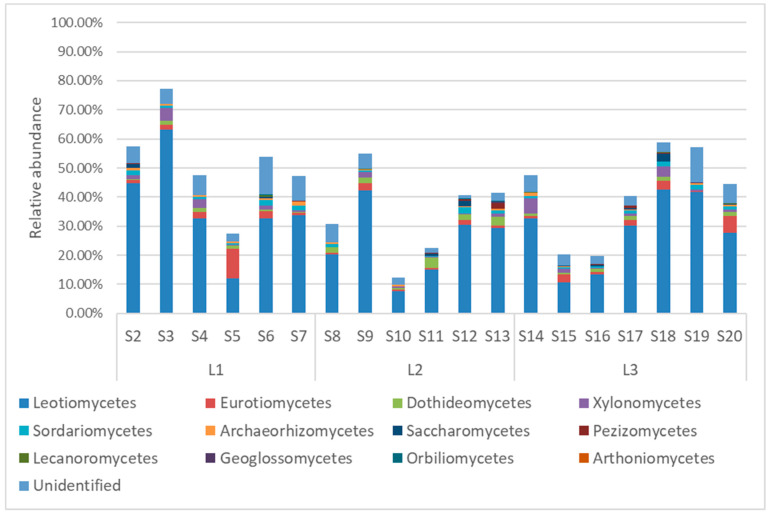
Soil fungal relative abundances at the class level of *Ascomycota* from soil samples taken from the Quimsacocha reserve, Ecuador.

**Figure 5:**
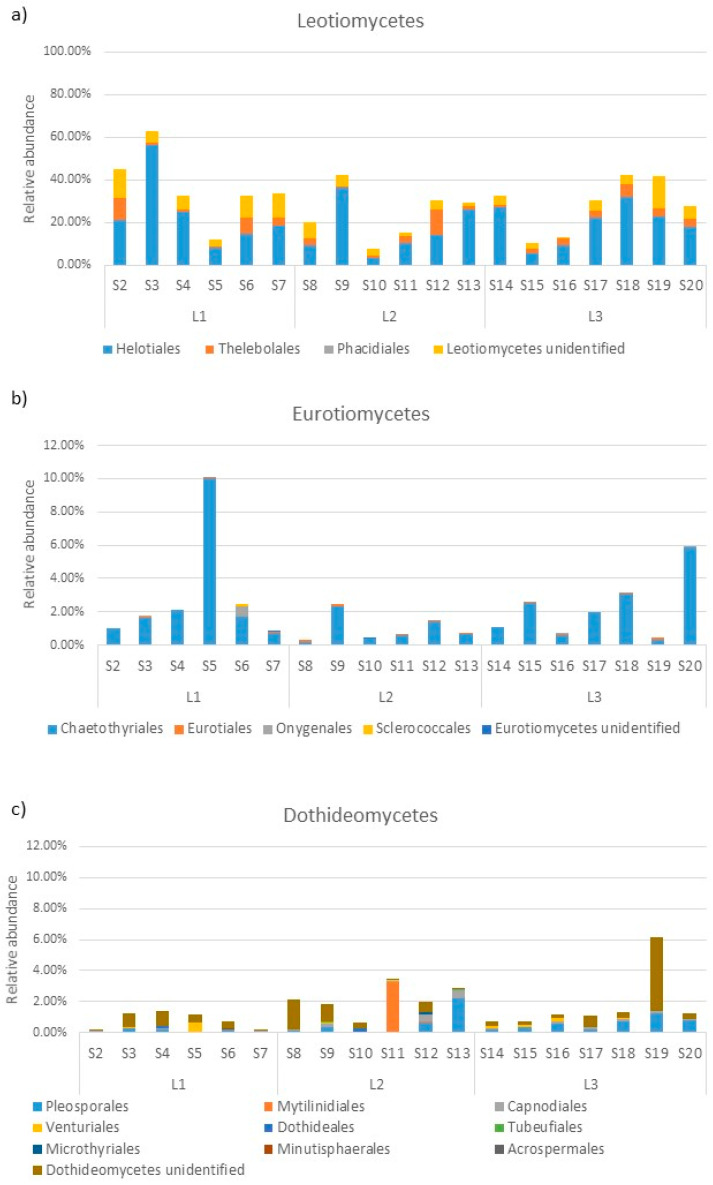
Order abundance in the most abundant classes of *Ascomycota*: *Leotiomycetes* (**a**); *Eurotiomycetes* (**b**); and *Dothideomycetes* (**c**).

Within the *Ascomycota*, *Leotiomycetes* was the dominant class in most samples, with an average abundance of 29.68%, ranging from 7.60% in S10 to 63.11% in S3 (Figure 4). This class was predominantly composed of the orders *Helotiales* (on average 20.18%) and *Thelebolales* (on average 3.64%, Figure 5a).

The classes *Eurotiomycetes* and *Dothideomycetes* were at approximately one order of magnitude lower in abundance than *Leotiomycetes* (on average 2.11% and 1.60%, respectively). The *Eurotiomycetes* were constituted mainly by the order of *Chaetothyriales* (ranging from 10% in S5 to 0.18% in S8), while the *Dothideomycetes* were constituted by nine different orders, among which *Pleosporales*, *Mytilinidiales*, and *Capnodiales* were the predominant ones (Figure 5b,c).

Among *Ascomycota*, 147 different genera were identified, with *Leohumicola* (on average 10.48%), *Microglossum* (on average 6.04%), *Pseudeurotium* (on average 3.05%), *Archaeorhizomyces* (on average 0.48%), and *Ramgea* (on average 0.44%) as the most abundant ones across all the samples (Supplementary Table S1).

##### Basidiomycota

**Figure 6:**
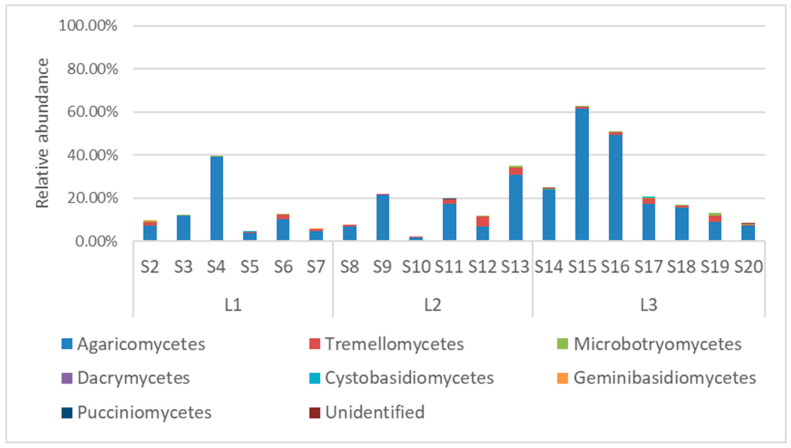
Soil fungal relative abundances at the class level of *Basidiomycota* from soil samples taken from the Quimsacocha, Ecuador.

The phylum *Basidiomycota* in the soil samples from Quimsacocha was mainly constituted by the class *Agaricomycetes* (on average 18.19%, ranging from 62% in S15 to 2% in S10, Figure 6). The only other class with an abundance higher than 1% was *Tremellomycetes* (on average 1.30%, ranging from 4% in S12 to 0.06% in S14). Another five classes were detected (*Microbotryomycetes*, *Dacrymycetes*, *Cystobasiodiomycetes*, *Geminibasidiomycetes*, and *Pucciniomycetes*), but their average abundance was lower than 0.3%.

**Figure 7:**
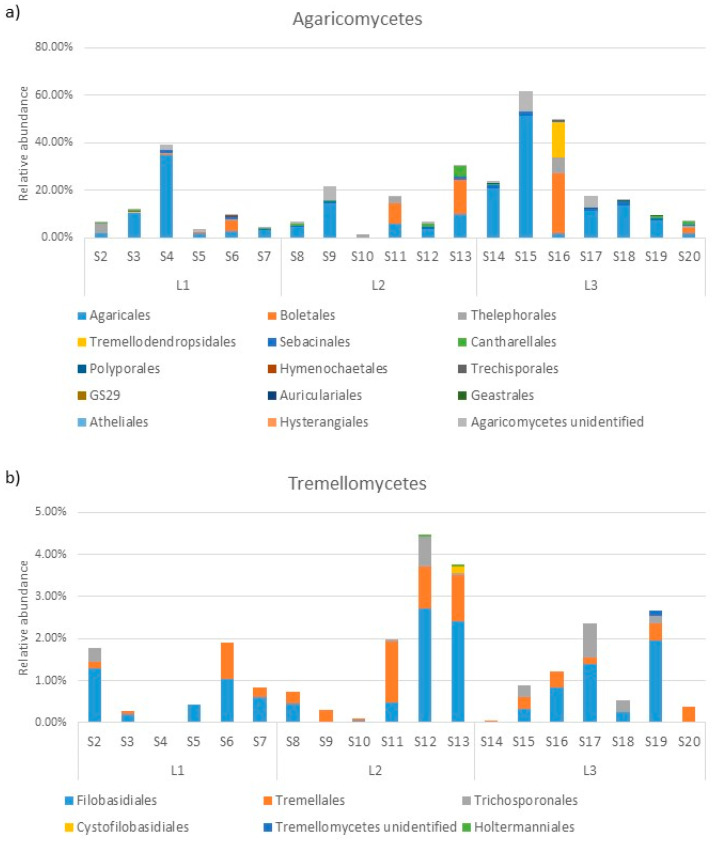
Order abundance in the most represented classes of *Basidiomycota*: *Agaricomycetes* (**a**) and *Tremellomycetes* (**b**).

Inside the class *Agaricomycetes*, the order composition varied among the samples. On average, the most abundant orders were *Agaricales* (10.64%, ranging from 51% in S15 to 0.29% in S10), *Boletales* (2.95%, ranging from 25% in S16 to being absent in samples S2, S3, S7, S8, S9, S14, S15, S17, S18, S19), *Thelephorales* (0.85%, ranging from 7% in S16 to being absent in samples S3, S7, S8, S9, S12, S14, S15, S17, S18, S19), and *Tremellodendropsidales*, which was a particularly abundant in S16 (15%) and absent in most other samples (Figure 7a). The class *Tremellomycetes* was constituted mainly by *Filobasidiales* (on average 0.76%, ranging from 3% in S12 to being absent in S4, S9, S14), *Tremellales*, mainly present in S11, S12, and S13, with an abundance around 1%, and *Trichosporonales*, mainly present in the samples S12 (0.69%) and S17 (0.81%, Figure 7b).

Among *Basidiomycota*, 67 genera were identified, with *Porpolomopsis* (on average 4.82%), *Rhizopogon* (on average 1.74%), *Suillus* (on average 1.22%), *Solicoccozyma* (on average 0.71%), and *Clavaria* (on average 0.59%) as the most abundant ones across all samples (Supplementary Table S1).

#### 3.2.2. Subkingdom Mucoromyceta

##### Mortierellomycota

**Figure 8:**
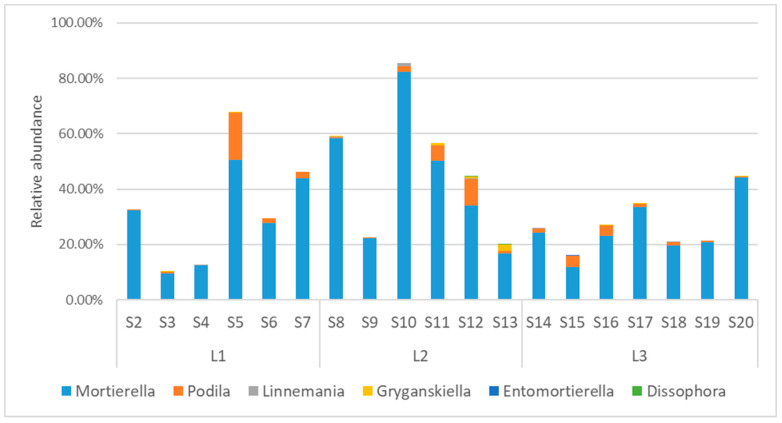
Relative abundance of genera in *Mortierellomycota*.

In the soils from Quisacocha, generally *Mortierellomycota* was the second most abundant phylum, showing a clear predominance in some samples (S5, S8, S10, S11, with an abundance of *Mortierellomycota* higher than 50%). Within this phylum, the most abundant genus was, by far, *Mortierella* (Figure 8). After that, there were the genera *Podila*, abundant in the samples S5 (17%), S12 (9%), and S11 (6%), *Linnemania* (present with an abundance higher than 1% just in S10), *Gryganskiella* (present with an abundance around 1% or higher just in S11 and S13), *Entomortierella* (only present in S15 with an abundance of 0.12%), and *Dissophora* (only present in S12 and S13, each with an abundance of 0.02%).

##### Glomeromycota

**Figure 9:**
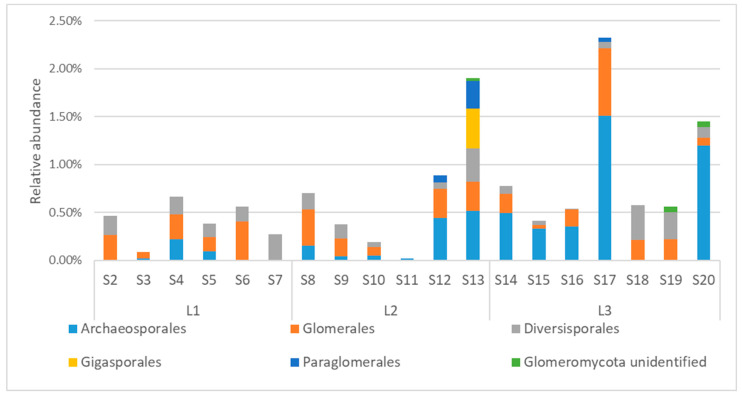
Relative abundance of orders in *Glomeromycota*.

Concerning the biodiversity of *Glomeromycota* in the soils of Quimsacocha, the taxa belonged to the orders of *Archeosporales* (on average 0.29%, ranging from 1.20% in S20, 1.51% in S17, to being absent in S2, S6, S7, S18, S19), *Glomerales* (on average 0.21%, ranging from 0.70% in S17 to being absent in S7), *Diversisporales* (on average 0.14%, ranging from 0.36% in S18 to being absent in S3, S11), *Gigasporales* (present only in S13 with an abundance of 0.42%), and *Paraglomerales* (present only in S12, S13, and S17, with an abundance of 0.07%, 0.29%, 0.04%, respectively, Figure 9). Unfortunately, a substantial portion of *Glomeromycota* was not identified at genus level, but the most abundant ASVs belonged to the genera *Ambispora*, *Acaulospora*, and *Archaeospora* (Supplementary Table S1).

##### Mucoromycota

**Figure 10:**
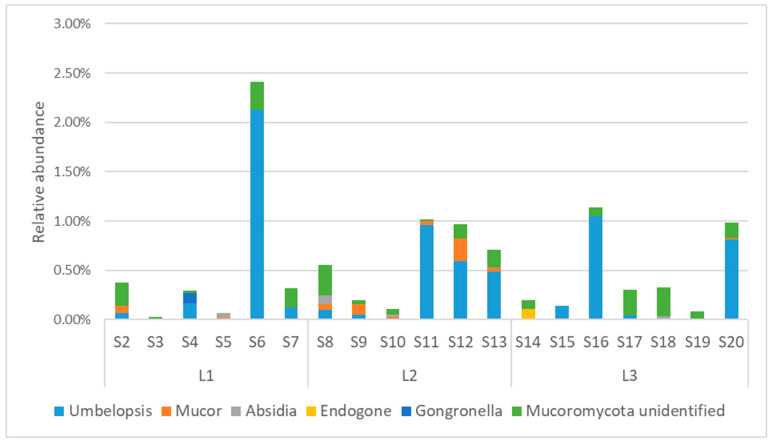
Relative abundance of genera in *Mucoromycota*.

The *Mucoromycota* fungi present in the soils of Quimsacocha belonged to the following three different classes: *Umbelopsidomycetes* (on average 0.36%), *Endogonomycetes* (on average 0.10%), and *Mucoromycetes* (on average 0.08%). At genus level, the composition of *Mucoromycota* in the samples is quite heterogeneous (Figure 10). The most abundant genus was *Umbelopsis*, which was particularly abundant in S6 (2.13%), S16 (1.05%), S11 (0.96%), and S20 (0.81%). The second most abundant genus was *Mucor*, and it was present with an abundance higher than 0.1% only in S9 and S12. The other identified genera were *Absidia*, *Endogone*, and *Gogronella*, but they were detected at very low abundances in every sample (Supplementary Table S1).

### 3.3. Soil Microbial Diversity in the Sampling Sites

**Figure 11:**
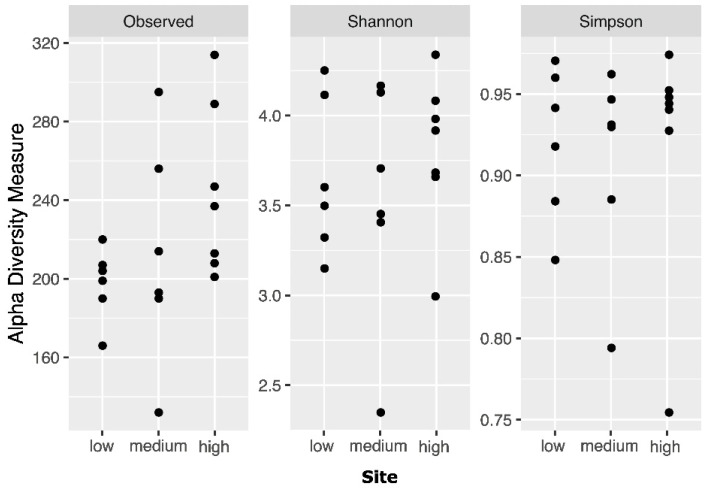
Estimated alpha diversity for the sampling sites at the three different altitudinal levels. No statistical differences were found between the three groups according the ANOVA test for each index. (**left**) Observed ASVs (*p* = 0.165); (**center**) Shannon (*p =* 0.657); (**right**) Simpson (*p* = 0.931).

The alpha diversity of the soil samples of Quimsacocha was quite variable, spanning from Shannon indices of 3.4 in S10 to 6.3 in S19 or Simpson indices of 0.75 in S15 to 0.97 in L6 and L19. This variability was also present within the three different altitudinal levels, and we could not find any significant differences among the alpha diversity indices between these groups (L1, L2, L3; ANOVA, *p* > 0.05; Figure 11).

**Figure 12:**
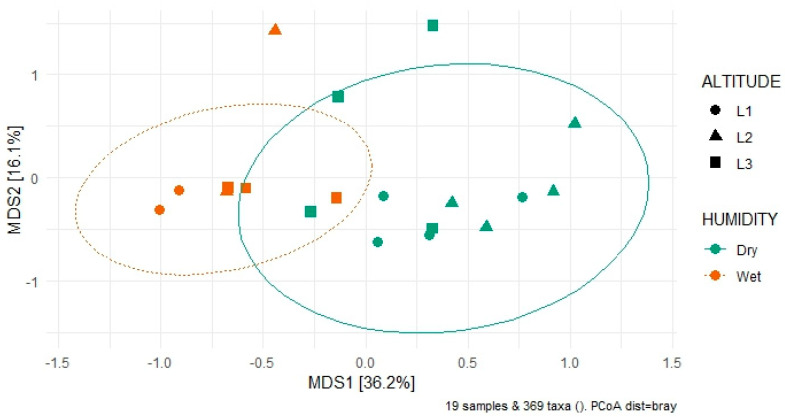
Principal Coordinates Analysis (PCoA) based on Bray–Curtis distances of soil fungal communities in Quimsacocha soils samples, divided according to their altitudinal level (ALTITUDE) or their moisture content (HUMIDITY).

Regarding the differences in fungal community composition, also known as beta-diversity, a PCoA ordination graph on Bray–Curtis distances was produced (Figure 12). The community composition of fungal assemblages did not vary significantly according to the three different altitudinal levels (PERMANOVA, *p* > 0.05). On the other hand, the moisture content of the samples influenced the community composition between the two groups of dry and wet soils (PERMANOVA, *p* < 0.001) significantly. Axis 1 and Axis 2 explained 36.2% and 16.1% of the total observed variance, respectively. Microbial communities with different moisture content clearly separated along Axis 1. These findings suggest that moisture content had, in the case of Quimsacocha samples, a stronger influence on microbial community composition than the altitudinal levels.

The differential abundance of fungal genera in dry and wet soil samples was calculated using a differential expression analysis (DESeq2 R package version 1.42.0) to understand which OTUs contributed to the differentiation of the two communities. Only three genera (*Clohesyomyces*, *Porpolomopsis*, and *Microglossum*) were detected as drivers of this dissimilarity, and all three were found to be significantly more abundant in wet samples.

## 4. Discussion

This study gives a thorough overview of bulk soil fungal diversity in an undisturbed area of the Andean Páramo in Ecuador. The soil samples, collected across three altitudinal levels and with different water contents, harbored very diversified fungal communities, suggesting that this area is a cradle for fungal diversity. The metagenomic analysis carried out identified the presence of 370 Amplicon Sequence Variants (ASVs) of fungi, mostly composed of *Ascomycota*, *Mortierellomycota* and *Basidiomycota*. To our knowledge, this work is the first study on the fungal diversity in the bulk soil of Ecuadorian Páramo.

The Páramo region of South America is still massively underexplored, especially considering fungal biodiversity. This tropical alpine ecosystem, extending itself on the Andes between 2900 and 5000 m.a.s.l., is often discontinuos, creating numerous unique ecological niches [28]. Most mycological studies on soil fungi in the Páramo focused on the Colombian portion [29,30,31], while the information on fungi in the Ecuadorian Páramo is scarce [32,33]. Pinos Leon et al. [32] described the microbial community in the rizosphere of the Andean blueberry, while Brück et al. [33] isolated and characterized some psychrotrophic fungi isolated from bulk soil.

In the analyzed soils of Quimsacocha, the fungal community composition and the ratio among taxa changed in each sample, even at phylum level. This could be an indication of the existence of several ecological niches for soil fungi in an exterior environment that can appear quite homogeneous. Future studies with an extensive sampling design and meticulous measurements of numerous environmental parameters could help shedding light on the reasons for this variability.

As also observed by Pinos Leon et al. [32] in the rhizosphere of the Andean blueberry in the Ecuadorian Páramo, the most abundant phylum was *Ascomycota*, and they detected also an abundance of *Mortierellomycota*. Moreover, all the four genera present in the core mycobiome of their rhizosphere samples, were detected also in this work: *Mortierella*, here present with an abundance of the 35%, *Solicoccozyma* (0.71%), *Cladosporium* (0.07%) and *Ilyonectria* (<0.01%), confirming their presence in the Ecuadorian Páramo from Quimsacocha.

Regarding the soil fungi found in the Páramo of other countries, only two other comparable works were made in Colombia [31,34]. Vélez-Martínez et al. [34] sampled soil from the Las Domínguez Regional Integrated Management District (Páramo, 3800 m.a.s.l.) and analyzed the soil fungal communities with metabarcoding. They found a similarly classed composition of *Ascomycetes* to what was detected in this work, featuring *Leotiomycetes*, *Archaeorhizomycetes*, *Dothideomycetes*, and *Sordariomycetes*. Moreover, there was an important correspondence also among the most abundant genera, as *Mortierella*, *Leohumicola*, *Pseudeurotium,* and *Archaeorhizomyces* were also detected by Vélez-Martínez et al. [34], suggesting their possible importance in the Páramo soils. Gualdrón-Arenas et al. [31] studied culturable fungi in the Páramo soil in the Special District of Santafè de Bogotà (Colombia) and managed to isolate numerous strains belonging to *Mortierella*, which is in accordance with our findings. Some other correspondences between taxa were found (*Epicoccum*, *Cladosporium*, *Mucor*, *Penicillium*, *Aureobasidium*, and *Fusarium*), but they were in low abundances in our study.

In the bulk soil fungal communities of Quimsacocha, *Mortierella* was the most represented genera, ranging from an abundance of 10% in S3 to 82% in S10. The genus *Mortierella* is mainly saprotrophic and ubiquitous in soil [35]. This finding confirms the abundance and importance of this genus in South America, especially in high altitude environments, even with very different vegetation covers [3,36].

*Leohumicola* was the second most abundant genus found in the samples of Páramo soils. This genus comprises seven different species and, even though its biogeography is understudied, it is thought to be a genus that dominates soil fungal communities around the world [37]. This genus is very tolerant to heat and its root-endophytism in roots of *Ericaceae* plants, quite abundant in the Quimsacocha study area [13], has some traits in common with ericoid mycorrhizae [38]. Species of the ascomycete *Microglossum*, detected with an abundance higher than 10% in six soil samples of Quimsacocha, usually have an earth tongue appearance, are often quite colorful, and they are common in undisturbed grasslands or open spaces [39]. To our knowledge, this is the first record of *Microglossum* in the Andean Páramo (it was found especially in the wet areas), and future mycological studies should focus on searching for this genus in the field to confirm this finding. Another interesting ascomycete found in these Páramo soils was *Archaeorhizomyces*, which is a genus of criptical, slow growing fungi commonly associated with plant roots [40]. They have been often detected in environmental DNA samples from soil and roots; however, since they are extremely difficult to isolate and culture, they remain understudied [41,42]. In addition to their association with plant roots, a sequencing study also suggested the involvement of *Archaeorhizomyces* in biological rock weathering and soil formation [43]. Interestingly, this genus was also found particularly abundant in the Colombian Páramo [4], suggesting an important role and association with Páramo plant roots which should be investigated further.

The presence of *Porpolomopsis*, especially abundant in wet soils of Quimsacocha, as highlighted by the differential expression analysis performed in this work, was quite unexpected. *Porpolomopsis* currently only includes two species. *P. calyptriformis* (Berk.) Bresinsky has mostly European distribution and represents the type species, while *P. lewelliniae* (Kalchbr.) Lodge, Padamsee and S.A. Cantrell is the main molecular reference and seems to be Australasian. Altogether, this opens two main questions. First, ASVs in the libraries may be perhaps referred to other taxa in *Hygrophoraceae* (such as *Hygroaster*) instead of *Porpolomopsis*; further analyses of the basidiome counterpart would help in striking at this concern, particularly in regard to elucidating what truly are the recurring taxa surveyed in Ecuador [3]. Second, if more evidence of *Porpolomopsis* occurrence in Neotropics was found, new conjectures on dispersal routes would be needed to explain the disjunctions in distribution. It should be finally observed that the *Porpolomopsis* species are generally non-invasive; *P. calyptriformis* has been declining [44], whereas *P. lewelliniae* is uncommon, even within its native range.

Besides *Porpolomopsis*, *Rhizopogon* and *Suillus* were the main basidiomycete genera in Páramo soils. As summarized by Mujic et al. [44], their host specificity in mycorrhizal symbiosis is the highest amongst all the known ectomycorrhizal fungi, and this trait is probably simplesiomorphic as they are phylogenetically very close with each other. Notably, *Rhizopogon* and *Suillus* are almost exclusively symbiont to *Pinaceae*, the former showing further subgenus-specific specializations. The *Rhizopogon* scenario reconstructed by Mujic et al. [44] is consistent with the lack of data from the humid climates in the Neotropics, since this genus likely evolved in northwest America and stopped its dispersal southward in Mexico—whereas it colonized Eurasia through the Beringian Land Bridge. *Pinaceae* are not native to South America, but biological invasion by introduced pines and their mycorrhizal partners *Rhizopogon* and *Suillus* has been widely documented in several areas [45]. Notwithstanding, it should be noticed that there is no record of introduced *Pinaceae* in Quimsacocha. Consequently, ambiguity in library references should be considered as a possible explanation for the *Porpolomopsis* case. Alternatively, these data may prelude to unexpected distribution patterns in *Boletales*.

These findings highlight the importance of the multi-method and critical approaches to biodiversity studies, particularly in poorly explored areas and habitats. Namely, the metabarcoding analysis of soils is just the first step to revealing the biodiversity of Quimsacocha, and future campaigns in the field could provide more data by surveying sporomes (for symbionts), visible forms (such as for biotrophic parasites), and culturable propagules (such as for saprotrophs).

Regarding the patterns of alpha and beta diversity, no difference was found in the three different altitudinal groups, even if some of the soil physico-chemical parameters measured in the three altitudinal levels varied significantly according to the altitude. This lack of statistical significance could be explained by the variability detected in the fungal community composition of the Quimsacocha samples. This could be an indication of the presence of different ecological niches in the soil environment shaped by other environmental factors. Indeed, the nearby presence of a source of water and the consequent high soil humidity level played a significant role in shaping the fungal community composition of Quimsacocha soils, which is understandable, especially because the Páramo in Southern Ecuador experience dry conditions. The driver of this change in the fungal communities was the increased presence of three fungal genera in wet soil samples, namely *Microglossum*, *Porpolomopsis*, and *Clohesyomyces* (Table 4). The fact that these genera are located preferentially in the wetter regions of the Quimsacocha Páramo is important information to retain for future sporome surveys and mapping.

This work represents a first glimpse at the studying of fungal biodiversity in an understudied ecosystem, the undisturbed Ecuadorian Páramo. Further studies will be needed to better appraise this diversity together with the development of necessary measures for environmental protection.

## Figures and Tables

**Table 1:** Sampling sites and number of soil samples.

Sampling Site	Number of Collected Samples	Altitude	Coordinate WGS 84 Datum UTM 17S	Area Ha.
L1	25	3040–3346	698,168.73–9,656,567.90	348.71
L2	25	3346–3656	696,462.02–9,657,059.22	1847.58
L3	27	3653–3960	696,670.66–9,658,961.80	5763.71

**Table 2:** Chemical properties of the soil. Values are expressed in ppm for each compound except Na; Mg, Ca, K are expressed in meq/100 mL; organic matter is expressed in % s.m.s (organic matter in dry weight); chlorine is expressed in meq/L. Common letter values in the same column are not significantly different (*p* > 0.05).

**Sampling** **Sites**	**Organic** **Matter**	**pH**	**NH_4_^+^**	**NO_3_^−^**	**P**	**K**	**Ca**	**Na^+^**
L1	54.17 ^a^	4.81 ^a^	143.30 ^c^	4.50 ^c^	14.49 ^a^	0.18 ^c^	1.41 ^c^	0.08 ^a^
L2	28.77 ^b^	4.70 ^b^	203.08 ^b^	9.10 ^a^	10.70 ^c^	0.34 ^a^	1.49 ^b^	0.03 ^b^
L3	25.79 ^c^	4.80 ^a^	272.11 ^a^	6.00 ^b^	10.80 ^b^	0.25 ^b^	1.99 ^a^	0.02 ^c^
**Sampling** **Sites**	**Mg**	**S**	**Zn^2+^**	**Cu^2+^**	**Fe^2+^**	**Mn**	**B**	**Cl^−^**
L1	0.51 ^b^	6.60 ^c^	7.40 ^a^	1.30 ^c^	880.80 ^a^	4.50 ^c^	0.01 ^a^	0.70 ^a^
L2	0.70 ^c^	8.81 ^b^	2.20 ^c^	3.03 ^b^	552.80 ^c^	5.30 ^b^	0.01 ^a^	0.70 ^a^
L3	0.72 ^a^	10.20 ^a^	4.50 ^b^	5.20 ^a^	716.20 ^b^	13.80 ^a^	0.01 ^a^	0.70 ^a^

**Table 3:** Soil moisture measurements. The values are expressed as a percentage (%) average and the standard deviation of three samples in each location. The dry group represents sampling sites where there were no nearby water sources and with an average water content lower than 55%; the wet group represents the sampling sites where nearby water sources were observed and had an average water content higher than 65%.

Altitude Level	Sample	Average % Humidity	Standard Deviation %Humidity	Humidity Group
L1	S2	45.3	1	Dry
	S3	76.1	0.2	Wet
	S4	81.6	0.2	Wet
	S5	51.1	0.9	Dry
	S6	49.4	0.3	Dry
	S7	42.8	2.7	Dry
L2	S8	39.4	0.3	Dry
	S9	71.4	0.9	Wet
	S10	51.6	0.4	Dry
	S11	42.2	0.3	Dry
	S12	47.1	0.2	Dry
	S13	68.6	0.6	Wet
L3	S14	72.1	0.5	Wet
	S15	37.5	0.1	Dry
	S16	51.3	0.2	Dry
	S17	67.8	1.5	Wet
	S18	78	0.8	Wet
	S19	48.5	0.3	Dry
	S20	75.2	1.1	Wet

**Table 4:** List of fungal genera with differential abundance in the samples divided according the humidity content (differential expression analysis based on the negative binomial distribution, *p*-values < 0.05, adjusted by false discovery rate).

Fungal Genera	Relative Abundance in Dry Soils	Relative Abundance in Wet Soils
*Microglossum*	0.09%	13.34%
*Porpolomopsis*	0.01%	10.36%
*Clohesyomyces*	0.00%	0.07%

## Data Availability

High-throughput sequencing data sets were deposited in the NCBI Biosamples database under accession numbers SAMN28020743, SAMN28020744, SAMN28020745 for Level 1, Level 2 and Level 3 for ITS DNA metabarcoding libraries, respectively.

## Supplementary Materials

The following supporting information can be downloaded at: https://www.mdpi.com/article/10.3390/jof10090623/s1, Table S1: from Fungal diversity in an undisturbed Andean Páramo soil of Quimsacocha (Ecuador) 2024.
